# DNA Vaccines against Dengue Virus Type 2 Based on Truncate Envelope Protein or Its Domain III

**DOI:** 10.1371/journal.pone.0020528

**Published:** 2011-07-11

**Authors:** Adriana S. Azevedo, Anna M. Y. Yamamura, Marcos S. Freire, Gisela F. Trindade, Myrna Bonaldo, Ricardo Galler, Ada M. B. Alves

**Affiliations:** 1 Laboratório de Biotecnologia e Fisiologia de Infecções Virais, Instituto Oswaldo Cruz, Fundação Oswaldo Cruz, Rio de Janeiro, Brazil; 2 Laboratório de Tecnologia Virológica, Instituto de Tecnologia em Imunobiológicos, Fundação Oswaldo Cruz, Rio de Janeiro, Brazil; 3 Laboratório de Biologia Molecular de Flavivirus, Instituto Oswaldo Cruz, Fundação Oswaldo Cruz, Rio de Janeiro, Brazil; Federal University of São Paulo, Brazil

## Abstract

Two DNA vaccines were constructed encoding the ectodomain (domains I, II and III) of the DENV2 envelope protein (pE1D2) or only its domain III (pE2D2), fused to the human tissue plasminogen activator signal peptide (t-PA). The expression and secretion of recombinant proteins was confirmed *in vitro* in BHK cells transfected with the two plasmids, detected by immunofluorescence or immunoprecipitation of metabolically labeled gene products, using polyclonal and monoclonal antibodies against DENV2. Besides, results reveal that the ectodomain of the E protein can be efficiently expressed *in vivo*, in a mammalian system, without the prM protein that is hypothesized to act as a chaperonin during dengue infection. Balb/c mice were immunized with the DNA vaccines and challenged with a lethal dose of DENV2. All pE1D2-vaccinated mice survived challenge, while 45% of animals immunized with the pE2D2 died after infection. Furthermore, only 10% of pE1D2-immunized mice presented some clinical signs of infection after challenge, whereas most of animals inoculated with the pE2D2 showed effects of the disease with high morbidity degrees. Levels of neutralizing antibodies were significantly higher in pE1D2-vaccinated mice than in pE2D2-immunized animals, also suggesting that the pE1D2 vaccine was more protective than the pE2D2.

## Introduction

Dengue is a mosquito-borne viral disease spread widely in tropical and subtropical regions in the world. It has been estimated that over 2.5 billion people live in areas of infection risk [1], [2]. In Brazil, where climatic conditions favor proliferation of the mosquito *Aedes aegypti*, the main vector of dengue viruses (DENV) [3], the disease was reintroduced in 1986 and since then dengue became a public health problem, with more than 7 million reported cases until 2010 [4], [5]. Virus infection can be asymptomatic or produce a broad spectrum of effects, including a mild febrile illness, the dengue fever (DF), or severe disease forms, the dengue hemorrhagic fever (DHF) and dengue shock syndrome (DSS) [6].

The DENV are enveloped viruses, which belong to the family *Flaviviridae*, genus *Flavivirus*, and consist of four antigenically distinct serotypes (DENV1-4) [7]. The virus genome is composed of a positive single-stranded RNA molecule, which codes a polyprotein precursor that is processed to produce three structural proteins, capsid (C), premembrane/membrane (prM/M) and envelope (E), and seven nonstructural (NS) proteins, NS1, NS2A, NS2B, NS3, NS4A, NS4B and NS5 [8]. The transmembrane E glycoprotein is the major component of virion surface and it is associated with numerous biological activities, since it acts as a binding protein, interacting with receptors present on host cell surface, and mediate membrane fusion of the envelope virus and host cell membrane [9], [10]. It is organized in 90 homodimers that become trimers at the fusion state [11]. Each monomer is composed of domains I, II and III, in which the domain III is responsible for the virus-cell interaction and trigger endocytosis of the viral particle [11]–[13]. Consequently, this protein is the main target for the induction of neutralizing antibodies, several of them against epitopes present in domain III [14]. In fact, most vaccines being developed against dengue virus are based on the induction of immune responses directed to the E protein from all four serotypes [15].

Several vaccine strategies have been proposed to control dengue disease, including classical approaches, as inactivated or live attenuated virus and subunit antigens, as well as new generation vaccines, such as chimeric engineered viruses and DNA immunization [10], [16]. The DNA vaccine approach has the advantage of *in vivo* antigen expression, leading to proper viral protein folding with post-translation modifications, such as N-linked glycosylation, which may be important for the induction of protective immune responses [17], [18]. DNA vaccines based on the E protein from dengue virus have been reported by several researchers [19]–[29], and most of them include prM and E gene sequences, since the prM seems to act as a chaperonin that may be necessary for correct envelope protein folding during virus exit from its assembly site through the Golgi system [27], [29]. However, several of these DNA vaccines induced low levels of neutralizing antibodies against dengue virus with consequently partial or short-term protection in different animal models [19], [21], [23], [27]. Moreover, most of these vaccines were constructed using the natural virus sequences that act as signal peptides for the secretion of these proteins, which may be not so efficient in the context of DNA vaccination.

Therefore, in the present work, we constructed two DNA vaccines encoding the ectodomain (domains I, II and III) of the DENV2 envelope protein (pE1D2) or only its domain III (pE2D2), fused to the human tissue plasminogen activator signal sequence (t-PA). For the expression of the ectodomain, corresponding to the 80% N-terminal protein sequence, the hydrophobic stem-anchor region of the envelop protein [30] was removed, in order to increase the efficiency of expression and secretion of the recombinant protein. On the hand, the t-PA sequence was used because of its efficiency in mediating secretion of recombinant proteins and antibody production in other DNA vaccines constructed by our group against dengue virus [31]–[33]. Results demonstrated that these plasmids were able to drive *in vitro* expression and secretion of recombinant proteins in mammalian cells. Both vaccines induced neutralizing antibody against DENV2 in Balb/c mice, although levels detected after immunization with pE1D2 were significantly higher than with pE2D2. In addition, all pE1D2-vaccinated mice survived challenge with a lethal dose of DENV2, while several animals immunized with pE2D2 died after virus infection or presented high morbidity rates.

## Materials and Methods

### Virus and Cells

The dengue 2 virus, strain New Guinea C (NGC DENV2), was used for the isolation of sequences coding fragments of the E protein and for challenge assays. The DENV2 44/2 [34] was used for PRNT_50_ assays. Virus propagation was carried out in Vero cells cultivated in Medium 199 with Earle salts (E199, Sigma, USA) buffered with sodium bicarbonate and supplemented with 10% fetal bovine serum (FBS, Invitrogen, USA). For the expression analysis of recombinant proteins *in vitro*, baby hamster kidney cells (BHK-21) were propagated in Dulbecco's Modified Eagle Medium (DMEM, Invitrogen), supplemented with 5% FBS.

### Construction of DNA vaccines

Two DNA vaccines were constructed encoding domains I, II and III of the E protein or only the domain III, named pE1D2 and pE2D2, respectively. These sequences were cloned in the pcTPA plasmid [31], a modified pcDNA3 vector (Invitrogen) which contains the human tissue plasminogen activator signal sequence (t-PA). Sequences were amplified by PCR using as template a previously constructed plasmid, pYFM/Nar, containing the full-length prM-E genes of the NGC DENV2 [34]. Sense and antisense primers, *5′GGGGGATATCATGCGTTGCATAGGAATATC3′* and *5′GGGGTCTAGATTAGATAGAACTTCCTTTC3′*, which anneals on the NGC DENV2 sequence (GenBank M29095), between nucleotides 937 to 956 and 2115 to 2130, respectively, were used for amplification of the E1D2 sequence, which corresponds to amino acids 1–398 of the E protein [35]. For amplification of the E2D2 sequence, encoding domain III of the E protein (amino acids 296–398), the sense primer 5′GGGGGATATCGGAATGTCATACTCTATG 3′, which anneals at the nucleotide 1822 to 1839 in the NGC DENV2 sequence, was used together with the antisense primer described above. Sense and antisense primers contained *Eco*RV and *Xba*I restriction sites, respectively. The PCR products were electrophoresed on a 1% agarose gel, recovered with glass beads, geneclean (Stratagene, USA), restricted with *Eco*RV and *Xba*I and ligated to the pcTPA previously digested with the same enzymes. All recombinant plasmids were screened by restriction mapping and confirmed by sequencing (ABI Prism dye terminator, Applied Biosystems, USA, performed by the Genomic Platform DNA Sequencing, PDTIS-Fiocruz). Recombinant plasmids were isolated from transformed *Escherichia coli*, DH5-α strain, and purified by Qiagen Plasmid Giga Kit (Qiagen, Germany), following manufacturer's instruction. DNA concentrations were determined by measuring optical density at 260 nm and integrity of plasmids was checked by agarose gel electrophoresis. Plasmids were suspended in sterile water and stored at −20°C until use.

### Cell transfection and Immunofluorescence assay

BHK cells were transiently transfected with the DNA vaccines pE1D2 and pE2D2 or the control plasmid pcTPA, as previously described [33]. Briefly, 2×10^4^ cells/well were plated in chamber slides (Nunc, Denmark) with Optimen medium (Invitrogen) and transfected with 0.2 µg of each DNA using lipofectamine (Invitrogen) under conditions suggested by the manufacturer. Cell monolayers were then maintained over night at 37°C with 5% CO_2_. In the following day, cells were washed in 0.1 M phosphate buffer pH 7.4, fixed in 4% paraformaldehyde for 10 min, permeabilized with 0.6% saponin for 10 min and blocked with 1% bovine serum albumin (BSA) and 0.2% saponin for 15 min. Cells were then incubated for 1 h at 37°C with DENV2 hyperimmune mouse ascitic fluid (ATCC, USA), diluted 1∶1500, or the monoclonal anti-DENV2 3H5 (ATCC), diluted 1∶500. Slides were then washed tree times and incubated for 1 h at 37°C with fluorescein-conjugated goat anti-mouse IgG (Southern Biotechnology, USA), diluted 1∶100, washed again and mounted with Vectashield medium (Vector Laboratories Inc., USA). Cells were visualized in a fluorescence microscope (Nikon Eclipse E600).

### Metabolic labeling and imunoprecipitation

For protein analysis, 5×10^5^ BHK-21 cells were transfected with 2 µg of each DNA as described above. In the following day, cells were metabolically labeled using [^35^S] methionine (80 µCi/mL) for 1∶30 h, in methionine-deficient RPMI 1640 medium (Sigma) without FBS. Culture supernatants were then immunoprecipitated with DENV2 hyperimmune mouse ascitic fluid, as previously described [36]. Protein samples were analyzed by 12% sodium dodecyl sulfate-polyacrylamide gel (SDS-PAGE) electrophoresis. Gel was dried and exposed to Kodak X Omat film for visualization of the labeled proteins.

### Immunization of Balb/c mice

Experiments with mice were conducted in compliance with ethical principles in animal experimentation stated in the Brazilian College of Animal Experimentation and approved by the Institute's Animal Use Ethical Committee (approval ID: L-067/08). Balb/c mice, 4 to 6 weeks old, were immunized by the intramuscular (i.m.) route with 100 µg of recombinant plasmids (50 µg in each tibialis posterior muscles), prepared in 100 µL of phosphate buffer saline (PBS) and using 27-gauge needles. Each mouse group (n = 10) received two doses of the DNA vaccine (pE1D2 or pE2D2) or the pcTPA vector, given 2 weeks apart. Two independent experiments were performed for each mouse group. Animals were bled by retro-orbital puncture, before inoculation (pre-immune sera), two weeks after the second immunization and 21 days after challenge, when all survived animals were sacrificed. Serum samples were prepared and stored at -70°C until use.

### Plaque reduction neutralization test (PRNT_50_)

Plaque reduction neutralization tests (PRNT) were carried out on Vero cells, in 96-well plates, as previously described [37]. Briefly, serum samples were serially diluted (from 1∶5 to 1∶640) in 50 µL of E199 medium followed by the addition of 50 µL of DENV2, corresponding to approximately 30 PFU, and incubated at 37°C for 1 h. Suspension of Vero cells were then added (2,5×10^4^ cells/well) and plates were incubated at 37°C for 3 h. After this period, media were discarded, cells were overlaid with 100 µL of E199 medium with 3% carboxymethylcellulose and plates were incubated for 7 days at 37°C in 5% CO_2_. Cells were then fixed with 10% formalin, stained with crystal violet and plaques were counted. Neutralizing antibody titers were expressed by 50% of plaque reduction (PRNT_50_).

### Mouse challenge with DENV2

Two weeks after the second DNA dose, mice were challenged with a mouse brain adapted sample of NGC DENV2. Animals were anesthetized with a mixture of ketamine-xylazine [38] and intracerebrally (i.c.) inoculated with 30 µL of 4.32 log_10_ PFU of DENV2, which corresponds to 3.8 LD50, diluted in E199 medium supplemented with 5% FCS. Immediately after challenge procedure, the inoculum was back-titered in Vero cells as described previously [34]. One group of non-immunized animals, challenged with DENV2, was also included as negative control. Animals were monitored for 21 days for mortality and morbidity, mainly the appearance of leg paralysis, alterations in spinal column and deaths. The evaluation of different morbidity degrees in each animal group was performed using a scale ranging from 0 to 3 (0 = none, 1 =  mild paralyses in one hind leg or alteration of the spinal column with a small hump, 2 =  severe paralyses in one hind leg and alteration of the spinal column with a small hump or severe paralyses in both hind legs, 3 =  two severe hind leg paralyses and deformed spinal column or death). Two independent challenge tests were performed in the same experimental conditions.

### Statistical analysis

Statistical analyses were performed using GraphPad Prism software (La Jolla, USA), version 5.02. For the analysis of survival and morbidity rates, statistical significances were evaluated by chi-square test, while differences in the degree of morbidity and PRNT_50_ titers were analyzed by Mann-Whitney test. Values were considered significant at p<0.05.

## Results

### Construction of the recombinant plasmids pE1D2 and pE2D2

**Figure 1:**
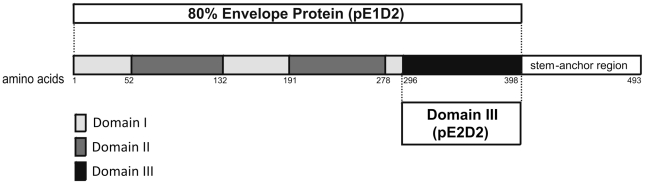
Schematic representation of the DENV2 envelope protein sequence. The protein is composed by the three domains (I, II and III) and the steam-anchor region (adapted from Modis *et al.* [12]). The sequence coding 80% of the N-terminal E protein, which contains domains I, II and III, was used for engineering the pE1D2 plasmid, while the pE2D2 plasmid encoded only the domain III sequence. Numbers indicated in the figure highlight amino acids corresponding the beginning and end positions at the primary protein sequence that compose each domain.

Two DNA vaccines (pE1D2 and pE2D2) were constructed encoding fragment sequences from the NGC DENV2 envelope protein. The plasmid pE1D2 contains the truncated sequence that encodes the first 398 amino acids, corresponding to domains I, II and III of the E protein, without the hydrophobic stem-anchor region, while the pE2D2 plasmid encodes only its domain III (from amino acid 296 to 398) (Fig. 1). Both fragments were cloned in frame with the t-PA signal sequence, which was used to target recombinant proteins into endoplasmic reticulum and its secretion to extracellular space.

### Analysis of the in vitro expression of recombinant proteins

**Figure 2:**
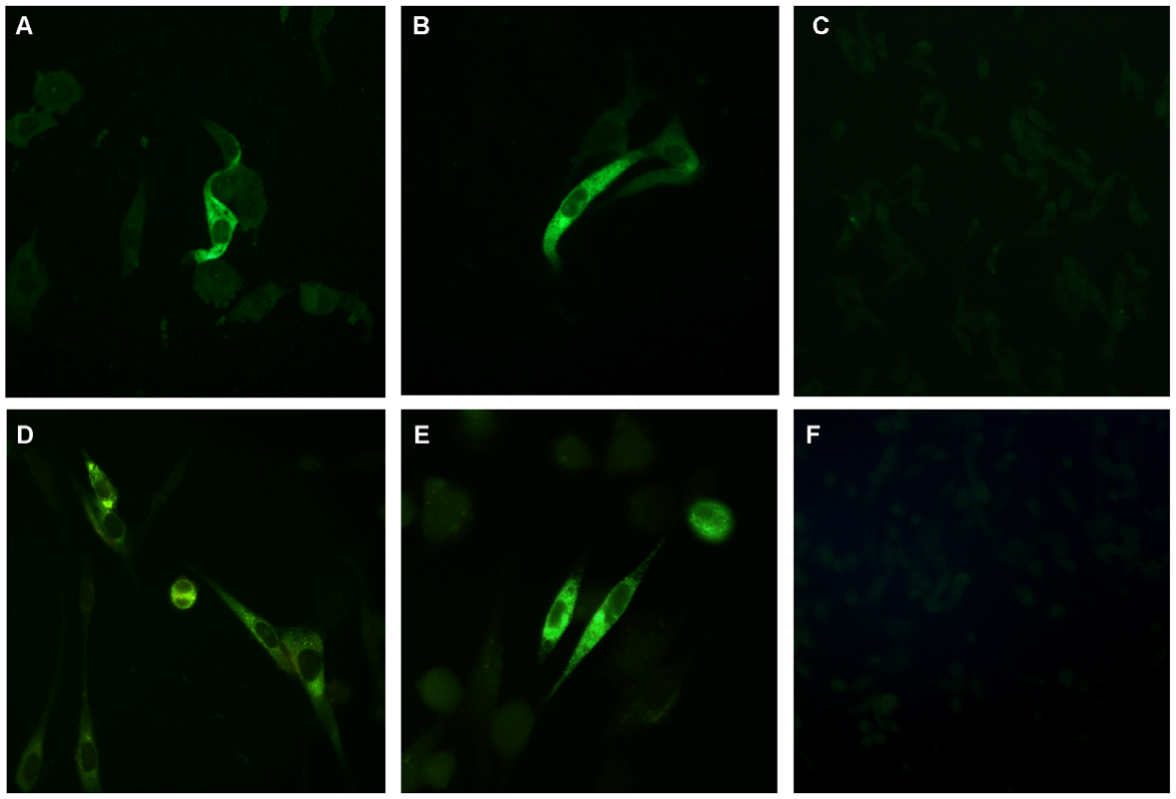
Analysis of the expression *in vitro* of recombinant proteins. BHK cells were transfected with plasmids pE1D2 (A, D), pE2D2 (B, E) and pcTPA (C, F). Cells were permeabilized, fixed and treated with DENV2 hiperimmune mouse ascitic fluid (A–C) or the monoclonal DENV2 3H5 antibody (D–F), followed by incubation with anti-mouse fluorescein-conjugated goat IgG. Magnification 1000x (A, B, D, E) and 400x (C, F).

The *in vitro* expression of recombinant proteins was evaluated in BHK-21 cells transiently transfected with the plasmids pE1D2 and pE2D2. Immunofluorescence assays revealed that cells transfected with these plasmids showed positive reaction with a hyperimmune ascitic fluid that recognized several epitopes of the DENV2 E protein during infection (Figs. 2A and 2B). The two recombinant proteins were also detected by the monoclonal 3H5 antibody, which is specific for a neutralizing epitope present on the domain III of the DENV2 envelope protein (Figs. 2D and 2E). As expected, no reaction was detected in control cells transfected with the pcTPA plasmid, using either the polyclonal or the monoclonal antibodies against DENV2 (Figs. 2C and 2F).

**Figure 3:**
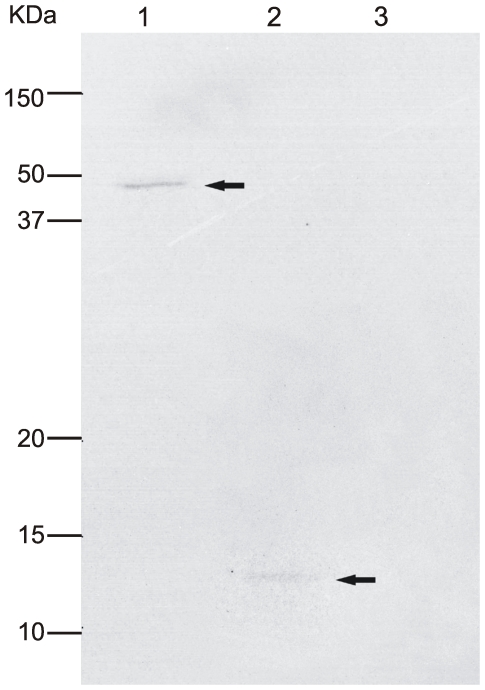
Electrophoretic analysis of recombinant proteins secreted by transfected BHK cells. Cells were metabolically labeled with [^35^S] methionine and culture supernatants were immunoprecipitated with DENV2 hiperimmune mouse ascitic fluid. Culture supernatants of cells transfected with pE1D2 (lane 1), pE2D2 (lane 2) or pcTPA (lane 3). Arrows indicate recombinant proteins.

The recombinant proteins were also observed in culture supernatants of transfected cells metabolic labeled, which were immunopreciptated with DENV2 hyperimmune ascitic fluid, thus revealing their secretion with expected molecular weights (approximately 44 KDa and 12 KDa for pE1D2 and pE2D2, respectively) (Fig. 3).

### Protective efficacy of the DNA vaccines in Balb/c mice

**Figure 4:**
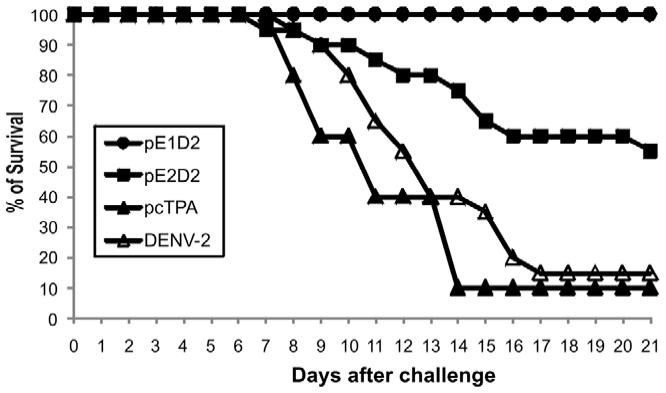
Percentage of survival of Balb/c mice immunized with pE1D2 and pE2D2 and challenged with DENV2. Mice were i.m. immunized with two DNA doses and challenged 4 weeks after the first plasmid inoculation. Non-immunized and pcTPA-injected mice followed the same virus infection procedure. Mice were daily monitored and deaths were recorded. Differences between pE1D2- and pE2D2-vaccinated animals were statistical significant (p = 0.0027), as well as between these groups and control animals (p<0.0001). Data represent compilation of two independent experiments, with groups of 10 animals in each test (n = 20).

**Figure 5:**
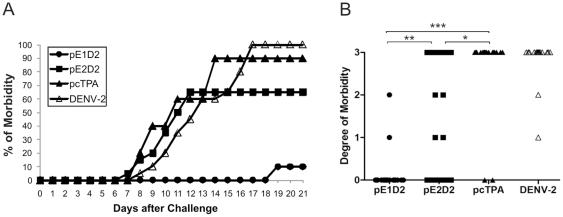
The morbidity in vaccinated mice after DENV2 challenge. The percentage (A) and degree (B) of morbidity of Balb/c mice immunized with pE1D2, pE2D2 and controls groups (non-immunized and pcTPA-inoculated animals) were analyzed after i.c. challenge with DENV2. Clinical signs of infection, mainly hind leg paralysis, alterations in spinal column and deaths, were monitored during 21 days post challenge (A). Differences in morbidity rates between pE1D2- and pE2D2-vaccinated mice were statistical significant (p<0.0001), as well as between pE1D2 and control animals (p<0.0001). The semi-quantitative analysis of morbidity degrees after virus challenge (B) were performed using a subjective scale ranging from 0 to 3 (0 = none, 1 =  mild paralyses in one hind leg or alteration of the spinal column with a small hump, 2 =  one severe hind leg paralyses and alteration of the spinal column with a small hump or two severe hind leg paralyses, 3 =  two severe hind leg paralyses and deformed spinal column or death). Asterisks indicate statistically significant differences between vaccinated animals and mice inoculated with the control vector pcTPA (* p = 0.0051; ** p = 0.0002; *** p<0.0001). Data represent compilation of two independent experiments, with groups of 10 animals in each test (n = 20).

Balb/c mice were immunized by the i.m. route with one of the two different DNA vaccines and challenged by i.c. inoculation with a lethal dose of a mouse brain adapted NGC DENV2. As control groups, non-immunized or pcTPA-inoculated animals were also challenged with DENV2. Animals were monitored the following 21 days after challenge for the development of leg paralysis, alterations in spinal column and death. Two independent challenge experiments were performed for each vaccine at the same conditions and data are summarized in figures 4 and 5.

All animals vaccinated with the pE1D2 plasmid survived challenge while 45% of mice immunized with the plasmid pE2D2 died after virus inoculation, and such differences were statistically significant (p = 0.0027) (Fig. 4). On the other hand, only 15% and 10% of animals in control groups (non-immunized or inoculated with the control vector pcTPA, respectively) survived after virus challenge (Fig. 4). All vaccinated animal groups, pE1D2 or pE2D2, presented significant differences in survival rates when compared to control groups (p<0.0001). Furthermore, control animals showed severe clinical signs of infection, starting at the 8^th^ day after challenge, and reached maximal levels of morbidity (100% and 90% in non-immunized or pcTPA-inoculated mice, respectively) in the following days (Fig. 5A). In contrast, only 10% of mice immunized with the pE1D2 vaccine presented clinical signs of the disease after the 19^th^ day post infection (Fig. 5A) with less severe morbidity degrees (Fig. 5B). Nevertheless, animals inoculated with pE2D2 presented clinical signs of infection from days 7 through 12 post challenge (65% of morbidity) (Fig. 5A). In this group, 45% of mice presented the highest severity of neurological signs (degree 3) and another 20% showed morbidity ranging from degree 1 to 2 (Fig. 5B). The differences between pE1D2- and pE2D2-vaccinated mouse groups were statistically significant, concerning either morbidity rates (p<0.0001) or the severity of the disease (p = 0.0002). However, both groups showed significant differences in the degree of morbidity comparing to control mice inoculated with the vector pcTPA (p<0.0001 and p = 0.0051, for pE1D2- and pE2D2-vaccinated animals, respectively).

### Neutralizing antibodies elicited by the DNA vaccines

**Figure 6:**
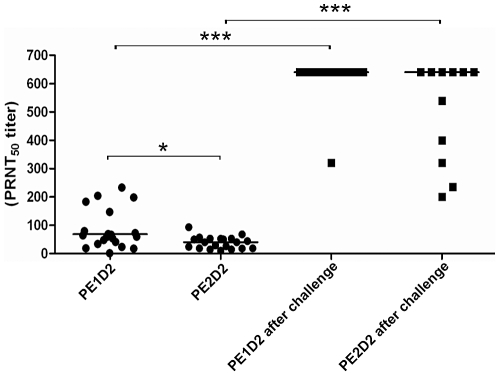
Plaque reduction neutralization test. The neutralizing antibody titrations (PRNT_50_) against DENV2 was evaluated in serum samples collected from pE1D2- and pE2D2-vaccinated mice (n = 20), before and after virus challenge. Individual samples were serially diluted from 1∶5 to 1∶640 and PRNT_50_ were performed in 96-well plates as described in material and methods. Asterisks indicate differences that are statistically significant between pE1D2- and pE2D2-immunized animals (*, p = 0.0029) or between vaccinated mice before and after virus challenge (***, p<0.0001).

The humoral immune response generated after immunization of Balb/c mice with the two DNA vaccines was analyzed by the presence of neutralizing antibodies. Serum samples from all vaccinated mice were collected two weeks after the second DNA dose, as well as from animals that survived challenge, obtained 21 days post infection, and titrated for neutralizing antibodies (PRNT_50_) using a different DENV2 isolate. Mice immunized with the pE1D2 vaccine presented significantly higher PRNT_50_ titers when compared to animals inoculated with the pE2D2 (p = 0.0029) (Fig. 6). Furthermore, the DENV2 virus challenge induced a remarkable increase in the levels of neutralizing antibodies in pE1D2-vaccinated mice, in which most of them (95%) reached the maximal neutralization titer used in this assay (1≥640). In contrast, pE2D2-vaccinated animals that survived virus infection (11 mice) presented a broad range of neutralizing antibody levels after challenge and only six of them (55%) showed maximum PRNT titers (Fig. 6).

As expected, pre-immune serum samples and sera collected from pcTPA-inoculated mice did not present detectable neutralizing antibody titers against DENV2 (data not shown).

## Discussion

In the present report we describe the construction of two DNA plasmids encoding the ectodomain (domains I, II and III) of the DENV2 envelope protein (pE1D2) or its domain III (pE2D2), fused to the t-PA signal peptide, in order to study the potential of such vaccines to induce antibody responses and protection in mice. The E protein was chosen since it is the major protein present on the surface of virus particle and it contains several epitopes that elicit neutralizing antibodies against DENV [39].

The expression of recombinant proteins was evaluated *in vitro* by the transfection of BHK cells with the two plasmids and detected by immunofluorescence assays. Results indicate that these DNA vaccines drove correct expression of such proteins in mammalian cells, since they were recognized by a polyclonal anti-dengue ascitic fluid that contains antibodies reacting to epitopes present on the DENV2 envelope protein during virus infection in different cells [27], [40], [41]. Furthermore, the proteins expressed by transfected cells were also recognized by the monoclonal antibody 3H5 [42], which is specific to a neutralizing epitope present on the domain III of the DENV2 envelope protein [43], thus revealing that such epitope is correctly presented in the recombinant proteins expressed by the two DNA vaccines. In the present study, plasmids were constructed with the t-PA signal sequence, which has already been used in other DNA vaccines and successfully mediated secretion of recombinant antigens with the induction of robust antibody responses [31]–[33]. Likewise, we observed secretion of the recombinant proteins mediated by both plasmids, pE1D2 and pE2D2.

Expression and immunogenicity of these proteins was then confirmed *in vivo*, by vaccination of Balb/c mice with the two different DNA vaccines, in which both plasmids were able to elicit neutralizing antibodies against DENV2. However, animals vaccinated with the pE1D2 plasmid presented significantly higher titers of neutralizing antibodies, when compared to pE2D2-inoculated mice. Although domain III in this case was immunogenic, since pE2D2-inoculated mice showed detectable levels of neutralizing antibodies, it seems not to be sufficient for the induction of an immune response with a magnitude similar to what was observed when we used larger segment of the E protein. Actually, the pE1D2 plasmid contains the sequence coding for the ectodomain of the E protein, which appears to be important for the generation of high levels of neutralizing antibodies, at least in the context of DNA vaccines. In fact, domains I and II also contain epitopes that generate antibodies which interfere with virus infection in cell culture [44], and therefore they could contribute for the highest neutralizing antibody levels detected in pE1D2-immunized mice. Moreover, Wahala and coworkers recently characterized the binding specificity and functional properties of human DENV immune sera and demonstrated that antibodies against domain III do not play the major role in DENV neutralization. Authors suggest, therefore, that other epitopes present on other locations in the E protein are primarily responsible for virus neutralization [45].

The protocol for mice immunization with DNA vaccine using the intramuscular route is a well established procedure that efficiently elicit robust immune responses in these animals, as shown by several authors [29], [31]–[33], [46], including our group with another plasmid encoding the DENV2 NS1 gene, in which the construction was similar to the pE1D2 and pE2D2 [31]–[33]. In general, the use of two doses of these DNA vaccines was already sufficient to induce desirable immune responses in mice [27], [31]. In the case of a DNA vaccine encoding the DENV2 NS1 gene, also fused to the t-PA signal sequence, animals inoculated with three plasmid doses presented only a transient increase in antibody levels, revealing a significant reduction in the following weeks, when they showed titers similar to those observed in mice immunized with only two DNA doses [31]. Moreover, it is always advantageous the use of a vaccine protocol with lower number of doses, especially in the case of immunizations against dengue in endemic regions, minimizing the risk of developing severe cases of the disease between vaccination intervals, in which a robust protective immune response is not completely achieved yet [47].

On the other hand, the ectodomain of the dengue envelope protein expressed in pE1D2 vaccinated mice, without the stem-anchor region, showed to be efficient for the induction of a protective immune response in mice after two DNA doses. The rationale for removing such region, which is localized on the C-terminal end of the protein, is because it is a highly hydrophobic amino acid sequence that may interfere with the expression and/or secretion of the protein, especially in heterologous systems. DNA vaccines based on truncated dengue envelope proteins were already developed for the induction of immune responses and protection against this virus [19], [22], [24]. All of these constructions used the dengue natural signal sequence present on the C-terminal end of the prM protein and none of them were fully protective, probably because of the lack of an efficient signal peptide for the context of DNA vaccines. On the other hand, some authors have used the t-PA sequence fused to the truncated E gene of Japanese encephalitis virus and they observed that such DNA vaccine conferred higher level of protection when compared to other tested plasmids [48].

Most of reported DNA vaccines against dengue and other flaviviruses are based on the full length of the prM and E genes [23], [27], [49]–[53], since it is believed that coexpression of both proteins is important for the correct folding of the E protein [8]. However, these proteins contain highly hydrophobic regions that may interfere with their expression and/or secretion [8]. In fact, some studies with DNA vaccines encoding prM/E sequences showed the induction of low titers of neutralizing antibodies and partial protection, even after several DNA doses [23], [46]. To overcome this difficulty, researchers employed other strategies such as the use of lysosomal targeting, immune stimulatory and/or cytokine sequences, prime/booster immunization regimen, etc [20], [25], [26], [28], [54]. Besides all such difficulty, recently Dejnirattisai *et al.* [55] showed that human monoclonal antibodies against the prM protein not only display limited virus neutralization capacity but may also enhance infection. Actually, it seems that the combination of partial cleavage of the prM, together with substantial cross-reaction between serotypes makes the anti-prM response particularly susceptible to enhancement of virus infection [55]. Therefore, the use of the envelope protein without the prM, as we did in the present work, may be more appropriate for the development of a vaccine against dengue virus, avoiding the risk of the increase of enhancement activities.

Immunization with the DNA plasmid encoding only the domain III (pE2D2), in its turn, induced low levels of neutralizing antibodies and incomplete protection against a lethal dose of DENV2. In contrast, other reports, using DNA vaccines encoding the domains III of the envelope protein from the four dengue serotypes, demonstrated different results [56], [57]. Ramanathan *et al*. [56] showed that such vaccines can induce humoral immune responses against all virus serotypes, with the production of neutralizing antibodies. However, in this study neutralizing antibody levels was not evaluated by PRNT_50_ assays, as performed in our work, and mice were not challenged with DENV in order to evaluate protection [56]. On the other hand, Mota *et al*. [57] using a combination of four DNA vaccines with the domain III of each virus serotype, have revealed protection induced by such vaccines. However, authors used indirect challenge models, by analyzing suckling mice inoculated with dengue viruses that were pre-incubated with immunized mouse serum samples, which may not reproduce what happens with adult animals [57]. In addition, the humoral immune response induced by such vaccines was of low magnitude, evaluated by ELISA, and the presence of neutralizing antibodies was not quantified [57].

The protective efficacy of our DNA vaccines was investigated by challenge tests with a lethal dose of a mouse brain adapted DENV2 isolate. Although such mouse challenge model, with i.c. virus inoculation, is quite distinct to what occur with humans, we choose it because it is the most widely used model for vaccines tests against dengue virus [19], [20], [22], [34], [58], [59] and, therefore, we could compare our results with those described in these studies.

In accordance to the levels of neutralizing antibodies observed in pE1D2-immunized mice, this animal group also showed high protective rates. Indeed, all Balb/c mice vaccinated with such plasmid survived challenge and only two animals (10%) presented some minor evidence of infection. On the other hand, protection conferred by the pE2D2 plasmid was only partial, since most of animals (65%) immunized with such vaccine showed clinical signs of infection after virus challenge and 45% of mice died in the following days. The pre-existing immunity in pE1D2-vaccinated mice is probably the basis for the observed protection, given the significant increase of antibody titers after virus challenge, which indicates the activation of immunological memory with rapid and strong secondary immune response, may have also contributed for such protection. However, we could not establish a direct correlation between morbidity degrees observed after challenge in animals immunized with the pE2D2 and their PRNT titers. Previous reports have shown that immunization with monoclonal antibodies against dengue virus with strong neutralizing activities *in vitro* induce different degrees of protection *in vivo* [60].

These studies suggested that the levels of neutralizing antibodies are not the only parameter to be investigated, since the stoichiometry of antibody binding may also be important for protection [60]. On the other hand, passive immunization of mice inoculated with sera collected from pE1D2-vaccinated animals revealed partial protection after virus challenge (34%), while the mean time to death was protracted (data not shown). Thus, our results suggest that, although neutralizing antibodies are important for protection against dengue virus, other branches of the immune response may also play a role in such protection. In fact, some reports have shown that cellular immune responses, including the activation of TCD4+ and TCD8+ cells, seem to be involved in protection against DENV [61]–[63].
